# The Histopathologic Examination of a Second Muscle Biopsy Specimen at a Later Date may Sometimes be the Best Approach to Make a Differential Diagnosis in Neuromuscular Disorders

**DOI:** 10.5146/tjpath.2019.01512

**Published:** 2022-01-21

**Authors:** Gulden Diniz, Berk Ozyilmaz, Sarenur Gokben

**Affiliations:** İzmir Demokrasi University, School of Medicine, Department of Pathology, Izmir, Turkey; University of Health Sciences, İzmir Tepecik Education and Research Hospital, Medical Genetics Lab., Izmir, Turkey; Ege University, School of Medicine, Department of Pediatric Neurology, Izmir, Turkey

**Dear Editor,**

Neuromuscular disorders still keep their mystery (1). Considering that cases with very mild symptoms cannot be diagnosed at all, it is almost impossible to know the true prevalence of these diseases (2). Relatively little information about the exact prevalence of neuromuscular disorders (NMDs) has been published (1–4). It has been reported that NMDs affect approximately one in 3500 children worldwide and X-linked dystrophinopathies have the highest incidence among them (4). Knowledge of NMDs has expanded dramatically during the last four decades thanks to advances in modern pathological techniques and genetic tests. Currently, the dystrophinopathies and most cases of limb-girdle dystrophies (LGMDs) can be diagnosed with immunohistochemical analysis of muscle tissues (4–7). It must be kept in mind that diagnoses may be suggested by the histopathological evaluation, but definitive diagnosis mostly relies on genetic analyses (5–7).

It can be thought that the evaluation by molecular techniques of comprehensive panels, each aimed at identifying genetic changes in a separate disease group, reduces the importance of pathological examination of muscle tissue (6). However, the evaluation of muscle biopsy specimens is an irreplaceable approach in some cases. For example, dystrophinopathy, the most common muscular disease, has two clinically and significantly different entities: Duchenne (DMD) and Becker muscular dystrophy (BMD). Among these two diseases that start in childhood, patients with DMD die in the second decade of their lives, and patients with BMD can reach their 50s thanks to close surveillance. Interestingly, genetic changes are similar in both diseases and cannot be distinguished by genetic analyses (4). The only way to differentiate the two diseases is to show the complete loss of dystrophin as in DMD or the presence of partially functional dystrophin protein as in BMD. Therefore, demonstration of sarcolemmal dystrophin in muscle biopsy tissue specimens has vital diagnostic importance (5). Similarly, the immunohistochemical examination of muscle biopsy specimens with commercially available primary antibodies is helpful in the diagnosis of many muscular dystrophies. In addition, staining of muscle biopsy specimens with combined succinate dehydrogenase-cytochrome oxidase (SDH-COX) enzyme stain is the most reliable way to diagnose mitochondrial myopathy in mitochondrial diseases with muscle involvement. Oil red O and PAS stains are also helpful for differential diagnosis of muscle involvement in metabolic diseases. In this way, unnecessary treatments can be avoided in many myopathies considered in the differential diagnosis (5–7).

Histopathologic evaluation of muscle biopsy specimens is an important step in the diagnosis of a neuromuscular disease. An experienced pathologist can provide very useful clues to the clinician for differential diagnosis. Presence of inflammatory cells or regenerated fibers, group atrophy, ragged red fiber, core or targets, increase in the number of central nuclei, accumulation of glycogen or lipid and fibrosis-like pathologies can be determined in neuromuscular disease. These findings discriminate against the disease group and determine the most useful genetic analysis that may be preferred for further examination. Especially in children older than 6-8 weeks, the presence of muscle fibers showing neonatal myosin expression in immunohistochemical analysis provides further evidence for a muscle disease (5–7).

Single muscle biopsy is almost always sufficient. For additional diagnostic approaches, the biopsy material stored at -80 oC should be re-evaluated by an experienced muscle pathologist with new dyes that have not been applied before. Repeat biopsy examination may be required only in newborn infants. In many muscle diseases, including DMD, recurrent biopsies only show a progression to an end-stage muscle disease and eventually the muscle tissue is replaced by fibroadipose tissue. There are few muscle diseases in which histopathologic examination of repeat biopsy specimens has diagnostic value. For example, in centronuclear myopathy, the nuclei in the muscle fibers are located peripherally at birth but migrate to the center in the following years, which is considered as a kind of dedifferentiation. Diagnosis cannot be made based on the histopathologic examination of the first biopsy specimens obtained in early childhood, and the diagnosis is usually established based on the examination of biopsy specimens harvested around the age of 4 (1–3).

**Figure 1:**
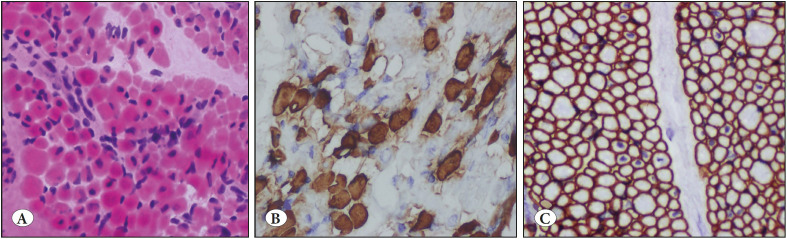
The first biopsy material. **A)** There are mild differences of myofiber size and a mild increase in internal nuclei (H&E; x100). **B)** The larger myofibers have type 2 features by anti-fast myosin antibody (IHC; x 100). **C)** Normal sarcolemmal dystrophin expression (IHC; x 100).

**Figure 2:**
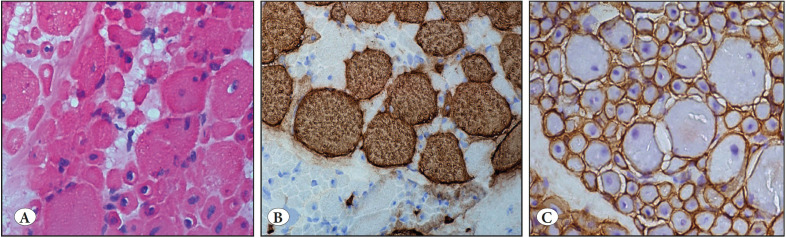
The second biopsy material. **A)** There are severe differences of myofiber size and most myofibers have internal nuclei (H&E; x200). **B)** The huge myofibers have type 2 features by anti- fast myosin antibody (IHC; x200). **C)** Normal sarcolemmal dystrophin expression and very prominent internal nuclei (IHC; x200).

A muscle biopsy specimen of a 10-month-old boy was evaluated histopathologically nearly five years ago. He was a hypotonic infant and had respiratory distress. There was no consanguinity between his parents. In the histopathologic examination of the first biopsy material, the presence of an increased number of central nuclei and myofibers of various sizes were detected. The patient was evaluated as a case of congenital myopathy such as centronuclear myopathy, myofiber-type disproportion and so on. In the first biopsy, there were mild differences in the size and shape of fibers. Numerous internal nuclei were not observed (Figure 1A-C). A repeat biopsy was suggested at a later date. Two years later, a new biopsy material was available. Interestingly, in the histopathologic examination of the second biopsy specimens, greater differences were seen in the sizes of fibers. Small fibers were of type1 and all fibers always had internal nuclei. Muscle injury had deteriorated to muscular dystrophy (Figure 2A-C). As the centronuclear myopathy must not have dystrophic features, and exaggerated differences in fiber sizes as seen in this case, the slides were consulted with an expert muscle pathologist, *Professor* Caroline Sewry, *Ph.D., FRCPath,* about the biopsy results who suggested a genetic evaluation. She wrote that central nuclei and small slow fibers seemed to be the main features, so a panel of congenital myopathy including MTM1, Titin (TTN), and RYR1 genes must be evaluated. Nearly a year later, we genetically evaluated these three genes and determined a homozygous MTM1 gene mutation (c.731C>T p.R241C). This variant was previously reported as pathogenic. Finally, the patient was diagnosed as a case of centronuclear myopathy caused by the mutation of the MTM1 gene on Xq28 (6).

In conclusion, unlike many other diseases, the histopathologic examination does not form the basis of the diagnosis of neuromuscular diseases. As in many parts of the world, a limited number of centers and a scarce number of trained pathologists are performing special muscle and nerve biopsy examinations in our country. Nevertheless, as observed in the present case, histopathologic examination, and more importantly, repeat biopsy is invaluable in the differential diagnosis. For this reason, it is very important to be able to perform the histopathologic examination of muscle and nerve biopsy specimens at least in some large centers and to provide the relevant training to the pathologists interested in this subject. 
